# Phenotypic profiling of pristane‐induced mimicking human systemic lupus erythematosus in *Macaca fascicularis*

**DOI:** 10.1002/ame2.70162

**Published:** 2026-03-15

**Authors:** Jonny Jonny, Soetojo Wirjopranoto, Chairul A. Nidom, I Ketut Sudiana, Terawan A. Putranto, Elisa D. Pratiwi, Tiza W. Mawaddah, Astria N. Nidom, Reviany V. Nidom, Setyarina Indrasari, Irma Y. Rosytania, Hiqmah Y. Yana, Sanindita Kusumastuti

**Affiliations:** ^1^ Doctoral Program of Medical Science, Faculty of Medicine Universitas Airlangga Surabaya Indonesia; ^2^ Indonesia Army Cell Cure Center Gatot Soebroto Central Army Hospital Jakarta Indonesia; ^3^ Faculty of Military Medicine Indonesia Defense University Bogor Indonesia; ^4^ Faculty of Veterinary Medicine Universitas Airlangga Surabaya Indonesia; ^5^ Department of Research and Development Laboratory Professor Nidom Foundation Surabaya Indonesia; ^6^ Global Biosains Teknologi Malang Indonesia; ^7^ Department of Pharmacy, Faculty of Health Sciences Adi Buana University Surabaya Indonesia

**Keywords:** animal models, *Macaca fascicularis*, pristane, systemic lupus erythematosus

## Abstract

**Background:**

The development of nonhuman primate models that replicate human systemic lupus erythematosus (SLE) remains limited. This study aimed to develop a pristane‐induced SLE model in *Macaca fascicularis* and evaluate its capacity to mimic human‐like clinical and laboratory immunological alterations.

**Methods:**

An experimental, single‐arm investigation was performed using six female *M. fascicularis* (2–3 years old, 3–4 kg), which received a single intraperitoneal pristane injection (5 mL/kg body weight) to induce SLE and were monitored biweekly.

**Results:**

Throughout the 24‐week study period, all macaques developed hallmark SLE‐like changes without requiring a booster, including a pronounced increase in antinuclear antibody titers (*p* = 0.002), with anti‐dsDNA positivity detected at the study endpoint. Significant decline was observed in hemoglobin, leukocyte, and lymphocyte count (*p* < 0.05), reflecting hematologic perturbations consistent with human SLE. Physiologic deterioration, manifested as hyperthermia and weight loss, also emerged early (*p* = 0.001). Biochemical assessment demonstrated mild hepatic and renal dysfunction marked by elevated serum glutamic pyruvic transaminase (SGPT) and urea concentrations (*p* < 0.05). Uniform proteinuria further indicated renal involvement, although the absence of hematuria suggests a spectrum of renal injury that may be less severe than that observed in advanced human SLE and may require longer observation.

**Conclusion:**

Overall, the reproducibility of autoantibody elevation and multisystem involvement demonstrates the model's translational potential. This nonhuman primate model offers a significant framework for investigating SLE pathogenesis and assessing novel therapy approaches.

## Graphical abstract

Pristane (5 mL/kg) induced a robust systemic lupus erythematosus (SLE)‐like phenotype in *Macaca fascicularis*, marked by elevated antinuclear antibody (ANA) levels and systemic clinical, hematological, and biochemical changes. This model closely reflects human SLE and provides a translational platform for disease studies and therapeutic evaluation. Created in BioRender. Jonny, J. (2025) https://BioRender.com/1sjj6hy.

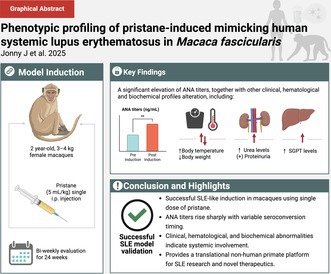

## 1 INTRODUCTION

Systemic lupus erythematosus (SLE) is an autoimmune disorder induced by genetic, immuno‐endocrine, and environmental influences. This syndrome results in tissue destruction through many pathways.^1^ The activation of T cells and B cells against self‐antigens induces the production of autoantibodies and the activation of the complement,^2^ all of which lead to tissue damage. The research and development of alternative treatments for the management of SLE remains in progress. To improve these treatments, we need animal models similar to human SLE. The development of SLE animal models is also important for a better understanding of its pathophysiology, genetics, and therapeutic strategies.

The use of multiple models is needed to evaluate drug efficacy on different genetic backgrounds and other mechanisms to provide a reference for clinical trials. Various SLE animal models have been developed: spontaneous model (NZB/NZW F1, MRL/lpr, BXSB), induced model, genetically modified model, and humanized model.^3^, ^4^ Pristane and imiquimod‐TLR7 were commonly used to induce SLE in animal models. Pristane is a hydrocarbon oil TMPD (2,6,10,14‐tetramethylpentadecane). It induces chronic inflammation in the intraperitoneal cavity. It also promotes the overproduction of interferon (IFN)‐α and ‐β.^5^ Adjuvant properties of pristane induce an autoantibody production. Many previous studies have applied pristane to induce SLE in animal models, such as in BALB/c mice^6^, ^7^ and C57BL/6J mice.^8^

The dominant animal model used for medication development in SLE is the mouse model. Nonetheless, assessments of pharmacological effectiveness and toxicity require an animal model exhibiting physiological parameters similar to those of humans. The complexity of the pathophysiology of SLE needs the application of nonhuman primates as an animal model. Currently, the advancement of SLE animal models in nonhuman primates is still limited. Recently, an SLE animal model in nonhuman primates was successfully developed using pristane induction. In that investigation, pristane was given at a dosage of 3.5 mL/kg of body weight, and the animal model was confirmed positive at week 27.^9^ Notably, the pristane induction required a second injection at week 17. It showed that the optimum dose remains unclear.

In this study, an SLE animal model was developed in *Macaca fascicularis* through a modified pristane induction protocol with a dosage different from that used in previous studies. The SLE phenotype was characterized based on antinuclear antibody (ANA) profiles, hematological parameters, and biochemical analysis of blood and urine samples.

## 2 MATERIALS AND METHODS

### 2.1 Study design

This is an experimental, single‐armed, pre‐post‐only study using *M. fascicularis*, a nonhuman primate model of pristane‐induced SLE. The experiment was conducted at the Animal Biosafety Level‐3 (ABSL‐3) Laboratory of the Professor Nidom Foundation (PNF Facilities), Surabaya, East Java, Indonesia.

### 2.2 Animals

A total of six female *M. fascicularis* aged 24 months and weighing 3–4 kg (CV Primaco Indonesia, West Java, Indonesia) were used in the study. All animals underwent an acclimatization period of 7 days prior to experimentation. During this period, animals were housed in a controlled laboratory environment with a temperature of 23 ± 1℃, relative humidity of 50% ± 10%, and a 12 h light/dark cycle. Standard primate diet and drinking water were provided ad libitum. Baseline health assessments were performed to ensure the animals met the inclusion criteria. All macaques were confirmed to be clinically healthy and specific pathogen‐free (SPF), defined as negative for simian immunodeficiency virus (SIV), influenza, and tuberculosis, with no record of previous experimental use. Macaques failing to meet this criterion were excluded from the study. Neither randomization nor allocation procedures were implemented in the experimentation as all macaques received the same treatment protocol. Potential confounders, such as handling order and sampling schedule, were kept constant among macaques to reduce environmental or procedural bias. Personnel responsible for daily husbandry and clinical monitoring were blinded to the study hypothesis but not to the intervention due to the visible nature of the procedure. During the final analysis, no macaques or data points were excluded. At the end of the study, all macaques were humanely killed with the assistance using ketamine (100 mg/kg body weight) and xylazine (4 mg/kg body weight).

### 2.3 SLE induction procedure

An SLE‐like phenotype in all macaques was generated by inducing an autoimmune response through the administration of pristane (≥98% pure, Sigma Chemical Co., USA), based on a predefined procedure prepared prior to the study. In this study, each animal received a single intraperitoneal injection of pristane at a dose of 5 mL/kg body weight, corresponding to a maximum volume of 15 mL. The development and progression of lupus‐like autoimmunity were longitudinally evaluated by assessing serum ANA titers at 2‐week intervals for 24 weeks.

### 2.4 Sample collection

Prior to blood collection, all animals were anesthetized with ketamine administered at a dose of 0.3 mg/kg body weight. For autoantibody, hematological, and biochemical assessments, 5 mL of whole blood was collected before the initial treatment and subsequently at 2‐week intervals over a 24‐week period. Collected blood samples were centrifuged at 1000*g* for 15 min to obtain serum, which was stored under appropriate conditions until further analysis. For urinalysis, urine samples were collected in the morning at the same time as blood samples to minimize diurnal variation.

### 2.5 Autoantibody assessment

ANA titer was the primary outcome of this study and was quantified using a Monkey ANA ELISA kit (FineTest, China). Whole blood samples were collected from the femoral vein and centrifuged at 1000*g* for 15 min to obtain serum. Samples were further analyzed using an ELISA kit according to the protocols provided by the manufacturer. ANA titer was calculated by substituting the optical density value at 450 nm (OD_450_) against the standard curve. Samples with ANA titer greater than or equal to 56.67 ng/mL were considered positive. To further confirm the establishment of SLE, serum anti‐double stranded DNA (anti‐dsDNA) antibody was assessed at the end of the study period using the ELISA kit (Elabscience, USA). Samples with an OD_450_ value greater than or equal to 0.199 were considered positive.

### 2.6 Hematological and biochemical assessment

Hematological and biochemical analyses were evaluated as the secondary outcome observed in this study. These analyses were performed concurrently with other examinations. Whole blood samples from each animal were subjected to complete blood count (CBC) testing, which included measurements of hemoglobin, total white blood cell (WBC) count, lymphocyte count, and platelet count. Serum obtained after centrifugation was analyzed for renal function markers (creatinine and urea) as well as hepatic enzyme activities, including serum glutamic pyruvic transaminase (SGPT) and serum glutamic oxaloacetic transaminase (SGOT).

### 2.7 Urinalysis

Fresh urine samples were collected in the morning at 2‐week intervals over a 24‐week period, as previously described. Urinalysis was performed using a commercial dipstick test in accordance with the manufacturer's instructions (Roche Diagnostic, Mannheim, Germany). Proteinuria and hematuria were assessed as indicators of renal involvement. Dipstick readings were evaluated visually using the provided color scale, with the intensity of color change corresponding to the relative concentrations of urinary protein and red blood cells. All assessments were carried out immediately after dipping to minimize inter‐observer variation.

### 2.8 Statistical analysis

All statistical analyses were performed using IBM SPSS Statistics software version 27.0 (IBM Corp., USA). Data were first examined for normality using the Shapiro–Wilk test to ensure the assumptions of parametric testing were met. Continuous variables are presented as mean ± standard deviation (SD). To evaluate the differences between pre‐ and post‐intervention measurements within the same group, a paired *t*‐test was applied. A *p*‐value of less than 0.05 was regarded as statistically significant. For anti‐dsDNA antibody, as assessments were obtained at a single time point, data were analyzed descriptively only to provide supportive serological evidence rather than comparative statistical inference. Graphical representations of the data were generated using SPSS and GraphPad Prism version10.0 (GraphPad Software, CA, USA) to illustrate mean changes and variability where applicable.

## 3 RESULTS

### 3.1 Clinical characteristics of induced SLE

After pristane administration, macaques in the study developed clinical features characteristic of lupus‐like disease. A significant reduction in body weight was observed (*p* = 0.001), accompanied by a marked elevation in body temperature (*p* = 0.001), suggesting the onset of systemic inflammatory response. Moreover, one macaque developed alopecia, which temporally coincided with the increase in ANA titers, indicating possible autoimmune‐related dermatologic involvement. No mortality was observed throughout the study period. Detailed clinical parameters are presented in Table 1, whereas longitudinal changes in body weight and body temperature for each macaque throughout the study period are shown in Figure S1.

**TABLE 1:** Comparison of clinical, hematological, and biochemical parameters in *Macaca fascicularis* before and after pristane‐induced SLE.

Parameter	Pre‐SLE (*n* = 6)	Post‐SLE (*n* = 6)	Mean difference	SE (95% CI)	*p*‐value
Body weight	3.23 ± 0.19	2.51 ± 0.41	−0.17 ± 0.33	0.13 (0.58–3.71)	0.001*
Body temperature	37.47 ± 1.07	39.22 ± 0.89	1.75 ± 1.34	0.55 (0.35–3.15)	0.001*

*Note*: Body weight is expressed in kg, whereas body temperature is expressed in°C. *p*‐value‐achieved significance is denoted in asterisk (*).

Abbreviations: CI, confidence interval; SLE, systemic lupus erythematosus.

### 3.2 Autoantibody analysis

After pristane administration, serum ANA titers markedly increased compared to baseline. The mean ANA titers increased from 36.88 ± 12.85 before induction to 61.13 ± 4.92 (*p* = 0.002). This significant elevation indicates the successful induction of systemic autoimmunity characterized by enhanced autoantibody production. These findings confirm the establishment of lupus‐like serological features after pristane administration, as shown in Table 2.

**TABLE 2:** Autoantibody titers following SLE induction.

	ANA (*n* = 6)	Mean difference	SE (95% CI)	*p*‐value
Pre‐SLE	36.88 ± 12.85	24.25 ± 12.18	4.97 (11.46–37.03)	0.002*
Post‐SLE	61.13 ± 4.92			

*Note*: ANA titer is reported in ng/mL. *p*‐value‐achieved significance is denoted in asterisk (*).

Abbreviations: ANA, antinuclear antibody; CI, confidence interval; SE, standard error; SLE, systemic lupus erythematosus.

**FIGURE 1:**
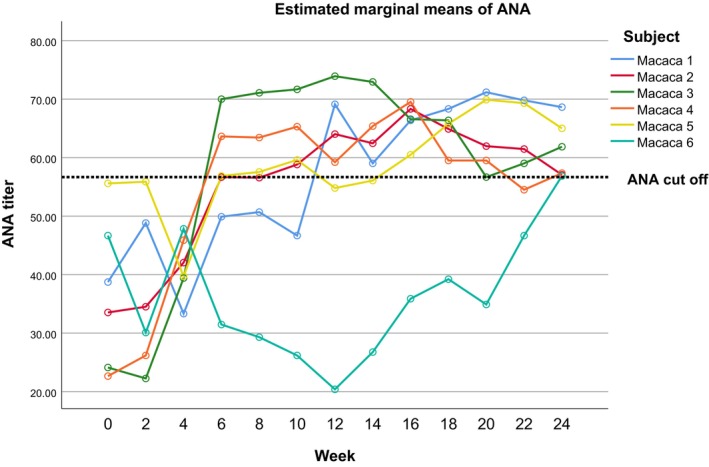
Antinuclear antibody (ANA) titer of six pristane‐induced *Macaca fascicularis* systemic lupus erythematosus (SLE) models. The autoantibody was evaluated every 2 weeks after pristane injection until 24 weeks.

In addition to the overall increase in ANA titers shown in Table 2, longitudinal monitoring revealed distinct individual patterns in the onset and fluctuation of antibody titers. ANAs were detected as early as week 6 in four of the six macaques after pristane administration, whereas the remaining two exhibited delayed seroconversion at weeks 12 and 24, respectively, indicating inter‐individual variability in autoimmune activation kinetic. Notably, Macaca 4 and 5, which developed early ANA positivity, experienced a transient decline below the cutoff at weeks 22 and 12, respectively, before titers increased again in subsequent weeks, as shown in Figure 1.

To further support the serological features of SLE, anti‐dsDNA antibody was assessed at week 24. Based on the predefined cutoff value of OD_450_ greater than or equal to 0.199, anti‐dsDNA positivity was observed in all six macaques. The OD_450_ values ranged from 0.199 to 0.306, with Macaca 4 having the lowest anti‐dsDNA level corresponding to the cutoff threshold. Notably, Macaca 6, which demonstrated ANA seroconversion at the end of the study period, showed the highest anti‐dsDNA OD_450_ value at 0.306. These findings indicate the presence of lupus‐associated autoantibody response at the late stage of disease induction. A graph illustrating inter‐individual variability in anti‐dsDNA levels at the study endpoint is provided in Figure S2.

### 3.3 Hematology parameters

Hematological alterations included significant reductions in hemoglobin, total WBC count, and lymphocyte count with *p*‐value less than 0.05, whereas platelet count showed a nonsignificant increasing trend (*p* = 0.089). Detailed hematology parameters are summarized in Table 3. The longitudinal trajectories of hematological parameters for individual animals are provided in Figure S3.

**TABLE 3:** Changes in hematological parameters following SLE induction.

Parameter	Pre‐SLE (*n* = 6)	Post‐SLE (*n* = 6)	Mean difference	SE (95% CI)	*p*‐value
Hemoglobin	9.62 ± 0.65	7.53 ± 1.29	−2.08 ± 1.52	0.62 (−3.68–(−0.48))	0.010*
WBC	7.38 ± 1.55	5.24 ± 1.34	−2.14 ± 1.46	0.62 (−3.71–(−0.58))	0.008*
Lymphocyte	2.88 ± 0.73	1.36 ± 0.37	−1.52 ± 0.65	0.26 (−2.21–(−0.84))	0.001*
Platelet	397.67 ± 149.70	533.00 ± 152.89	135.33 ± 211.88	86.49 (−87.02–357.68)	0.089

*Note*: Hemoglobin is reported in g/dL, whereas WBC, lymphocyte, and platelet counts are expressed in ⨯10^9^ cells/L. *p*‐value‐achieved significance is denoted in asterisk (*).

Abbreviations: CI, confidence interval; SE, standard error; SLE, systemic lupus erythematosus; WBC, white blood cells.

### 3.4 Biochemical analysis

Biochemical analysis revealed a mild but significant elevation in SGPT (*p* = 0.035) and urea levels (*p* = 0.013), suggesting early hepatic and renal involvement, whereas SGOT and creatinine changes were not significant. Proteinuria was observed in all post‐induction subjects (*p* = 0.002), despite the absence of hematuria in all macaques throughout the study period, further supporting renal manifestation of SLE. To be precise, proteinuria was found in all macaques between weeks 20 and 24, reflecting progressive renal manifestation over time; however, one macaque exhibited the resolution of proteinuria by the end of the study period. The absence of hematuria in all animals suggests that overt glomerular injury was not detectable by dipstick urinalysis during the experimental period. Detailed data for all measured parameters are presented in Table 4, whereas longitudinal trends of biochemical parameters and urinalysis findings are shown in Figure S4.

**TABLE 4:** Changes in biochemical analysis and urinalysis following SLE induction.

Parameter	Pre‐SLE (*n* = 6)	Post‐SLE (*n* = 6)	Mean difference	SE (95% CI)	*p*‐value
Liver function					
SGOT	67.48 ± 35.13	62.72 ± 32.79	−4.76 ± 39.29	16.04 (−46.00–36.47)	0.389
SGPT	25.77 ± 23.43	43.03 ± 19.88	23.25 ± 24.91	10.17 (−2.89–49.40)	0.035*
Renal function					
Creatinine	0.98 ± 0.26	0.76 ± 0.29	−0.23 ± 0.44	0.18 (−0.69–0.24)	0.132
Urea	16.48 ± 6.17	26.83 ± 5.04	10.35 ± 8.04	3.28 (1.91–18.79)	0.013*
Proteinuria	0	62.5 ± 30.62	62.50 ± 30.62	12.50 (30.37–94.63)	0.002*

*Note*: SGOT and SGPT are reported in U/L. Creatinine, urea, and proteinuria are reported in mg/dL. *p*‐value‐achieved significance is denoted in asterisk (*).

Abbreviations: CI, confidence interval; SE, standard error; SGOT, serum glutamic oxaloacetic transaminase; SGPT, serum glutamic pyruvic transaminase; SLE, systemic lupus erythematosus.

## 4 DISCUSSION

The current study successfully established a human SLE‐like condition in *M. fascicularis* using pristane induction. This finding supports the translational applicability of pristane‐induced models beyond murine, demonstrating that nonhuman primates can also recapitulate key immunopathological characteristics of human SLE. Pristane‐induced inflammation is toll‐like receptor (TLR)‐dependent, resulting in the production of IFNs,^10^ the stimulation of B cells to make autoantibodies,^11^ and a decreased ability to remove apoptotic cells.^12^ Pristane stimulates TLR7, then activates plasmacytoid dendritic cells (pDC) and macrophages to produce IFNs,^13^ a pathway consistent with the broader role of dendritic cells in coordinating innate immune activation and immune regulation in systemic autoimmunity, including SLE.^14^ This innate response through pristane continues to autoantibody production and immune complex formation.^15^ When injected into the peritoneal cavity, pristane causes a localized inflammatory response, mimicking important immunopathological characteristics of SLE.^16^

The production of autoantibodies targeting DNA,^17^ histones,^18^ nucleosomes,^19^ and other nuclear constituents facilitates the development of immune complexes.^16^ It induces the release of pro‐inflammatory cytokines. In addition to IFNs, the other cytokines, including IL‐6,^20^ IL‐17, and TNF‐α,^21^ are elevated under SLE conditions. Moreover, pristane promotes the lipogranuloma formation, further magnifying the inflammatory response.^22^ These pathophysiological processes cumulatively result in the malfunction of several important organs, such as the kidneys, liver, lungs, and spleen, as indicated by modifications in organ function tests and histological alterations.^23^ In the current investigation, hepatic and renal dysfunctions were shown, mirroring those observed in human SLE, marked by increased SGPT and urea concentrations. Furthermore, the appearance of proteinuria corroborated renal impairment.

Interestingly, three biochemical parameters, including SGOT, creatinine, and platelet count, showed nonsignificant reduction after SLE induction. Consistent with the findings in this study, another experiment using a murine model also reported no increase in SGOT levels, mirroring the pattern of liver involvement typically observed in human SLE.^24^ SGOT is known to be expressed in various non‐hepatic tissues, including skeletal muscle and myocardium, whereas SGPT serves as a more specific marker for hepatocellular injury.^25^ This difference may explain the significant elevation of SGPT in the current study compared with SGOT, which showed no significant difference after SLE induction.

Similarly, decreased serum creatinine levels were also reported by Krishnamurthy et al.^26^ in 2023, suggesting that creatinine may have limited sensitivity for detecting early renal involvement in SLE, as it is easily influenced by dietary protein intake, catabolic status, or gastrointestinal loss. This finding aligns with previous reviews indicating that significant renal injury may occur before measurable impairment of renal function.^27^ During the early phase of renal involvement in SLE, compensatory hyperfiltration of surviving nephrons can preserve glomerular filtration rate, allowing serum creatinine to remain within the normal range despite ongoing renal damage.^28^

This may be further supported by oxidative stress analysis in pristane‐induced lupus in murine models, which showed no significant alterations in antioxidant enzyme activities up to 4 months post‐induction, with notable reductions only after 6 months, suggesting that early disease stages may not yet involve renal injury, allowing serum creatinine to remain stable despite SLE.^29^ This highlights a critical limitation in relying solely on creatinine‐based monitoring in both experimental and clinical lupus nephritis, underscoring the need for more sensitive early biomarkers of renal inflammation.^30^ In line with this, in this study, proteinuria was observed in all subjects between weeks 20 and 24, reflecting progressive renal manifestation over time, whereas hematuria remained undetected throughout the experiment.

A similar trend was observed in platelet count, which showed a slight increase rather than decrease typically associated with advanced disease in SLE. Compared to other hematological abnormalities such as anemia, leukopenia, and lymphopenia, which were common in SLE, thrombocytopenia has been reported in only approximately 38% of patients in a recent study of human SLE.^31^ This observation is consistent with findings from the pristane‐induced lupus murine model developed by Chapacais et al.^32^ in 2025, which demonstrated a significantly higher splenic megakaryocyte proliferation as part of an enhanced extramedullary hematopoietic response. The increased megakaryocyte activity has been proposed to represent an early compensatory response that causes platelet production to be transiently upregulated in response to pro‐inflammatory cytokines, before immune‐mediated platelet destruction happens in later disease stages.^33^ Pro‐inflammatory cytokines, particularly IL‐6 and type I interferons, have been shown to be involved in this process by promoting thrombopoiesis, in part through the upregulation of hepatic thrombopoietin (TPO) synthesis, a pathway shown to be essential for IL‐6–mediated platelet elevation.^34^ Although IL‐6 was not directly measured in this study, substantial evidence from previous studies supports its role in inflammation‐driven thrombopoiesis and platelet alteration in SLE, providing a plausible mechanistic framework for the transient platelet elevation observed. Overall, the transient elevation in platelet count observed in this study may likely reflect an inflammatory as well as compensatory hematopoietic state, rather than an abnormal laboratory finding.

The ANA titer is a key diagnostic indicator for SLE.^35^ This study revealed an overall increase in ANA titers following pristane induction, with marked inter‐individual variability in the time of onset among *M. fascicularis*. Notably, delayed ANA seroconversion did not correspond to the milder disease phenotype. Instead, the macaque that demonstrated a delayed ANA seroconversion, becoming ANA‐positive only at week 24, exhibited more pronounced clinical and biochemical alterations compared to the others, particularly in parameters that showed significant changes, suggesting a temporal dissociation between autoantibody emergences and disease manifestation. This observation mirrors clinical SLE, that the macaque pristane‐induced model can capture inter‐individual heterogeneity in disease onset and severity, closely resembling the clinical diversity observed in human SLE, a feature that is often limited in murine models.^36^ The detection of anti‐dsDNA antibodies at the study endpoint further supports the establishment of lupus‐associated autoimmunity, as anti‐dsDNA represents a disease‐relevant biomarker linked to immune dysregulation and disease activity.^37^ Collectively, these findings are consistent with prior research demonstrating that pristane‐induced SLE requires CD38 and TLR7 for the initiation of local inflammation and autoantibody production.^16^ It denotes the complex interaction between innate immune activation and adaptive immunological dysregulation in the pathogenesis of SLE.

To date, only two studies have reported the induction of SLE using pristane in nonhuman primates. The first was conducted by Wang et al.,^9^ who established a pristane‐induced lupus‐associated model in *M. fascicularis* using a dose of 3.5 mL/kg body weight administered intraperitoneally on two occasions with a 17‐week interval. Although this protocol successfully induced lupus‐like features, including autoantibody production and renal involvement, it required repeated dosing and was associated with a relatively prolonged latency before disease manifestations became evident. In contrast, the present study used a single intraperitoneal administration of a higher pristane dose (5 mL/kg body weight) without booster injections. This regimen resulted in earlier ANA seroconversion, consistent hematological abnormalities, and biochemical evidence of organ involvement within a shorter observation period. Compared with widely used murine SLE models, including MRL/lpr or NZB/W F1 mice as well as the emerging humanized SLE mice, which provide valuable mechanistic insights but face translational challenges including limited reconstruction of the human B cell immune response and restricted lifespan that limits long‐term disease observation,^38^, ^39^ the nonhuman primate pristane‐induced model offers improved translational relevance. From an experimental and translational perspective, the single‐dose approach may offer advantages in terms of procedural simplicity, reproducibility, and reduced experimental burden for preclinical therapeutic evaluation. Nevertheless, direct comparisons between dosing strategies remain limited, and future studies incorporating dose–response and booster‐comparison designs are warranted to further refine the pristane‐induced nonhuman primate SLE model.

Furthermore, the use of *M. fascicularis* enhances the translational relevance of this model, as their immunological system, disease progression, and systemic responses more closely resemble human SLE compared with rodent models. Several limitations of this study should be acknowledged. The small sample size inherent to nonhuman primate experiments may limit statistical power and preclude extensive subgroup analyses. In addition, cytokine profiling, including IL‐6, was not directly assessed, limiting mechanistic interpretation of the observed hematological and inflammatory alterations. Nevertheless, the present study provides a comprehensive characterization of a single‐dose pristane‐induced SLE model in *M. fascicularis*, capturing inter‐individual heterogeneity and clinically relevant serological and biochemical features. Future studies incorporating longitudinal cytokine measurements, immune cell phenotyping, and extended follow‐up periods in larger study will be necessary for wider generalization.

## 5 CONCLUSION

The autoantibody titer, hematology profile, and organ dysfunction observed in this study show that a single dose of 5 mL/kg per body weight was an effective single‐dose induction strategy to induce human SLE‐like in *M. fascicularis*. The average onset of SLE occurred as early as week 6, with the latest seroconversion occurring at week 24, without the need for booster pristane administration. Hence, an animal model that closely recapitulates key clinical, serological, and biochemical features of human SLE may be valuable for advancing the understanding of disease pathophysiology and supporting preclinical therapeutic development. This model may therefore serve as a robust preclinical platform for studying disease heterogeneity and evaluating immunomodulatory interventions prior to clinical translation.

## AUTHOR CONTRIBUTIONS

**Jonny Jonny:** Conceptualization; methodology; resources; supervision; writing – original draft; writing – review and editing. **Soetojo Wirjopranoto:** Supervision; validation. **Chairul A. Nidom:** Conceptualization; project administration. **I Ketut Sudiana:** Supervision; validation. **Terawan A. Putranto:** Conceptualization; supervision; validation. **Elisa D. Pratiwi:** Data curation; formal analysis; investigation; software. **Tiza W. Mawaddah:** Data curation; formal analysis; investigation; software. **Astria N. Nidom:** Data curation; investigation; project administration; software. **Reviany V. Nidom:** Data curation; investigation; project administration. **Setyarina Indrasari:** Data curation; methodology; project administration. **Irma Y. Rosytania:** Methodology; supervision. **Hiqmah Y. Yana:** Formal analysis; software; visualization; writing – original draft. **Sanindita Kusumastuti:** Formal analysis; software; visualization; writing – original draft.

## FUNDING INFORMATION

This study was conducted without external funding.

## CONFLICT OF INTEREST STATEMENT

All authors declare that they have no competing interests.

## ETHICS STATEMENT

The animal study protocol was approved by the Institutional Animal Care and Use Committee (IACUC) of Professor Nidom Foundation (approval number: 021224/IACUC/VII/2024). All procedures were conducted in accordance with relevant institutional and national guidelines for the care and use of laboratory animals.

## PERMISSION TO REPRODUCE MATERIAL FROM OTHER SOURCES

All figures and tables included in this manuscript are original and were generated by the authors.

## Supporting information


Data S1.

## Data Availability

The data presented in this study are available on reasonable request from the corresponding author.
