# Supplementary material for: Phenotypic profiling of pristane‐induced mimicking human systemic lupus erythematosus in Macaca fascicularis
Source: Animal Model Exp Med. 2026 Mar 15;9(7):1302–9. doi: 10.1002/ame2.70162 (PMC13393505; doi:10.1002/ame2.70162)
Supplement: Supplementary file 1 — Data S1. [file AME2-9-1302-s001.docx]

**Supplementary Materials**

**
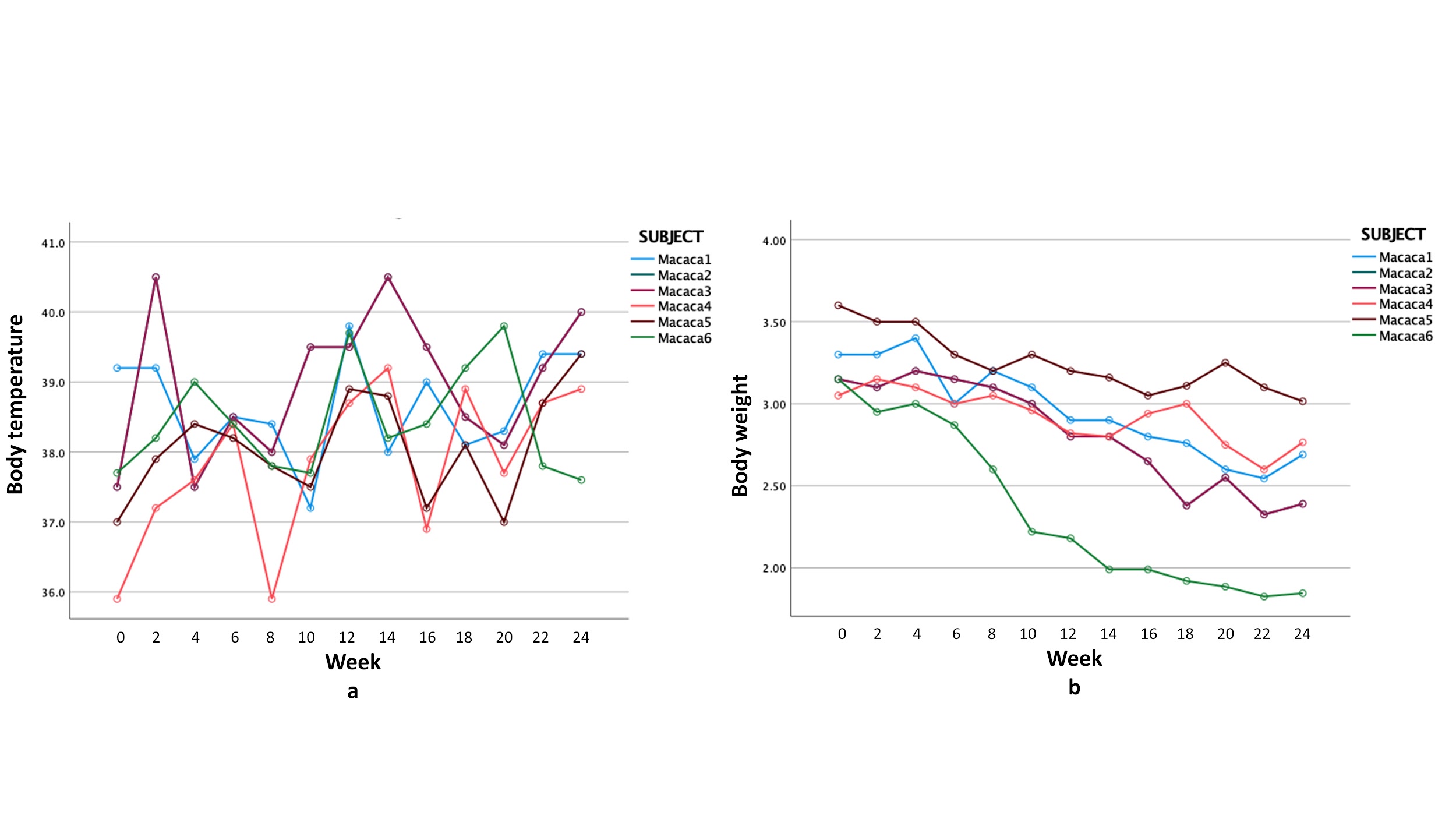
**

Figure S1. Longitudinal changes in body temperature (a) and weight (b) for each macaque throughout the study period. Body temperature is presented in ℃, while body weight is presented as kilogram (kg).


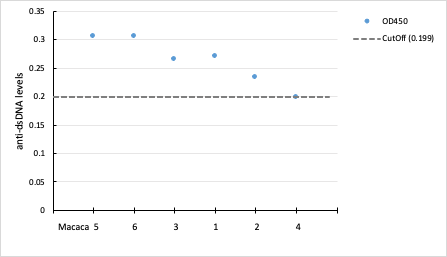


Figure S2. Graph showing individual anti-dsDNA antibody levels measured at week 24 following pristane administration. Each dot represents one macaque. The dashed horizontal line indicates the predefined cut-off value (OD_450_ = 0.199) for anti-dsDNA positivity.

**
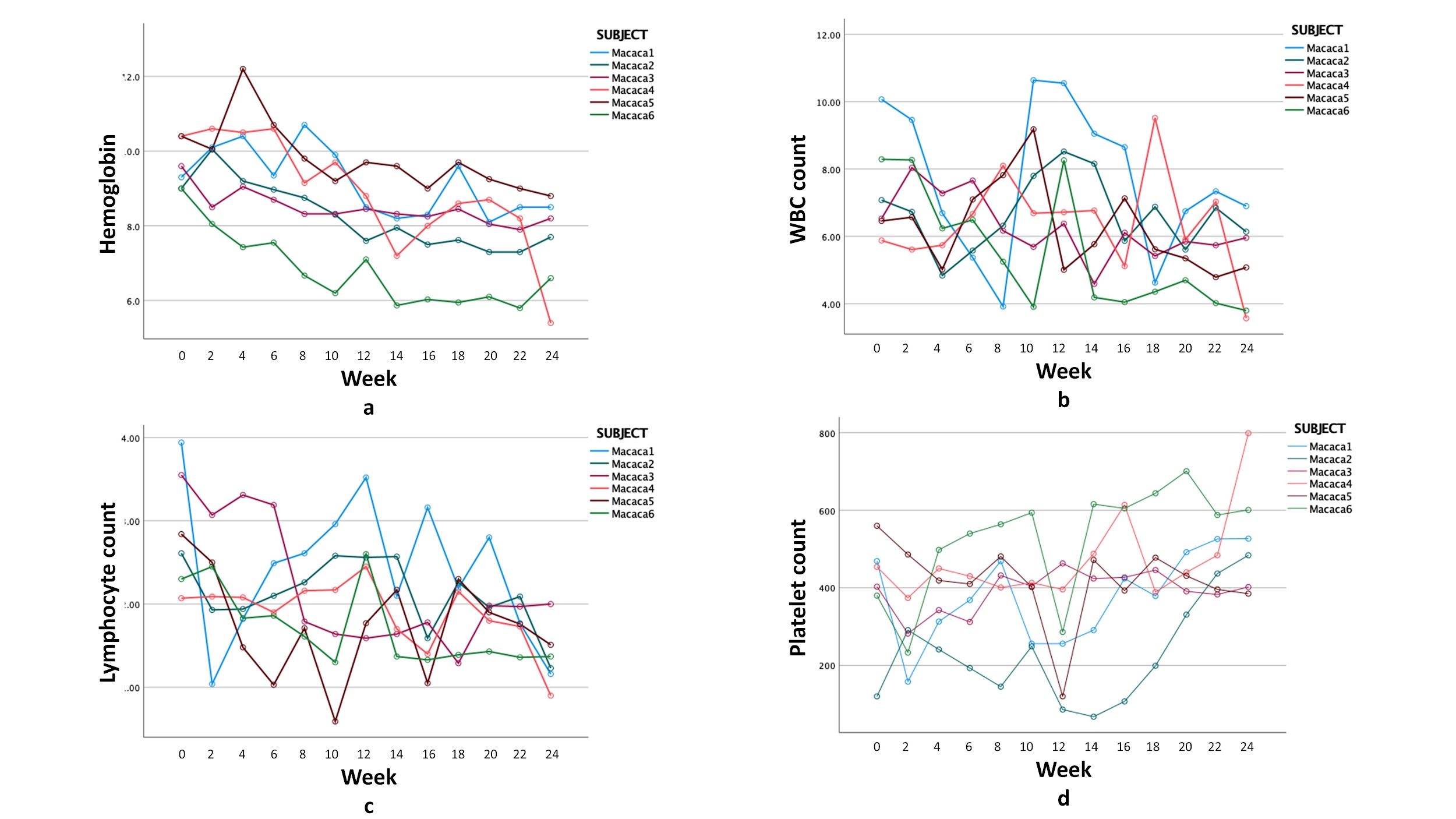
**

Figure S3. Longitudinal changes in hematological parameters for each macaque throughout the study period. Hemoglobin levels (a) are expressed in g/dL, whereas WBC (b), lymphocyte (c), and platelet count (d) are expressed as ⨉10^9^ cells/L.


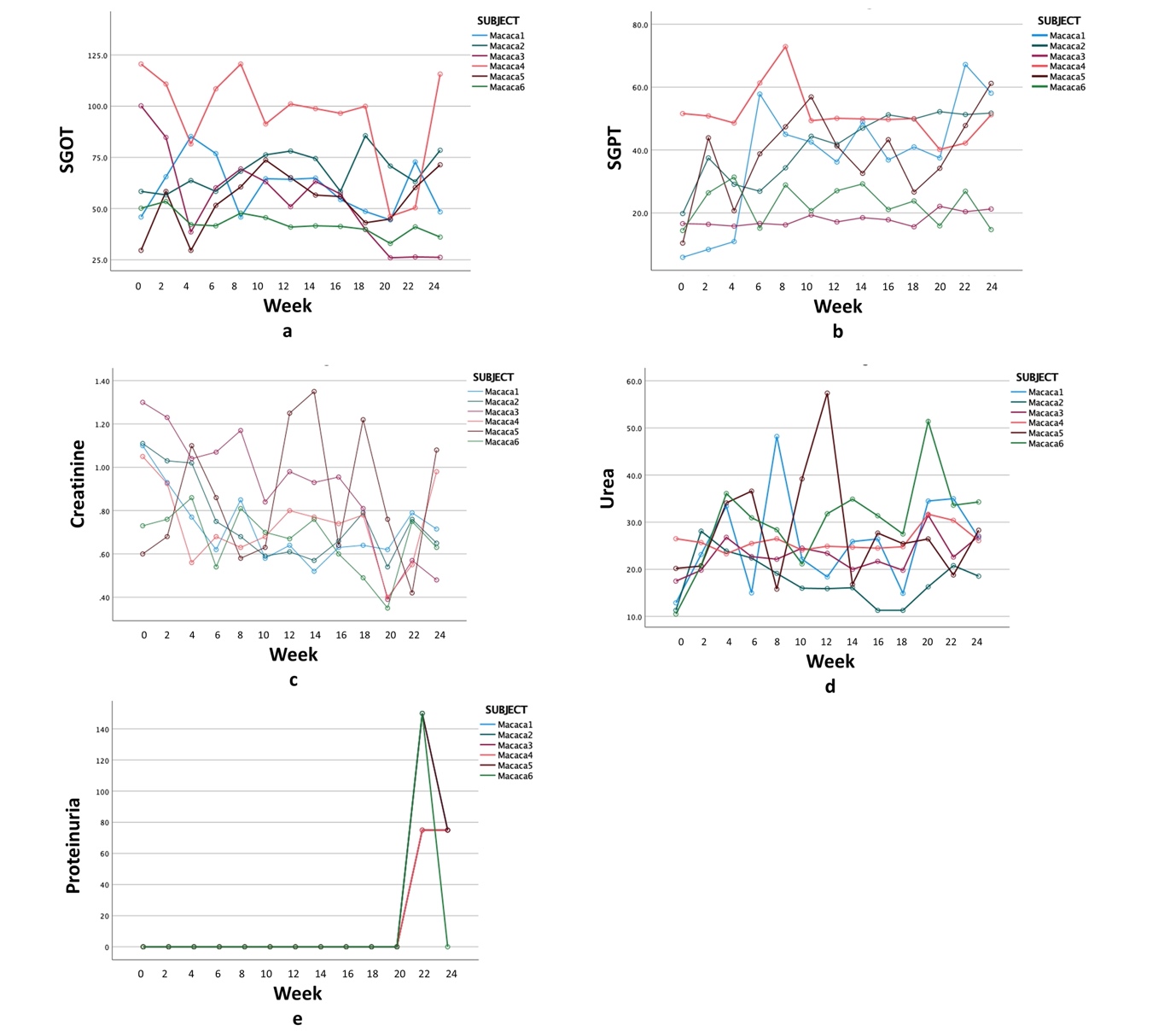


Figure S4. Longitudinal trends of biochemical parameters and urinalysis findings for each macaque throughout the study period. SGOT (a) and SGPT (b) are reported in U/L, while creatinine (c), urea (d), and proteinuria (e) are reported in mg/dL.
